# Complete Genome Sequencing of a Novel Type of *Omikronpapillomavirus 1* in Indian River Lagoon Bottlenose Dolphins (Tursiops truncatus)

**DOI:** 10.1128/genomeA.00240-18

**Published:** 2018-04-26

**Authors:** Thaís C. S. Rodrigues, Kuttichantran Subramaniam, Galaxia Cortés-Hinojosa, James F. X. Wellehan, Terry Fei Fan Ng, Eric Delwart, Stephen D. McCulloch, Juli D. Goldstein, Adam M. Schaefer, Patricia A. Fair, John S. Reif, Gregory D. Bossart, Thomas B. Waltzek

**Affiliations:** a Department of Infectious Diseases and Immunology, College of Veterinary Medicine University of Florida, Gainesville, Florida, USA; b Department of Small Animal Clinical Sciences, College of Veterinary Medicine, University of Florida, Gainesville, Florida, USA; c Department of Comparative, Diagnostic, and Population Medicine, College of Veterinary Medicine, University of Florida, Gainesville, Florida, USA; d College of Veterinary Medicine, University of Georgia, Athens, Georgia, USA; e Blood Systems Research Institute, Department of Laboratory Medicine, University of California, San Francisco, California, USA; f Department of Laboratory Medicine, University of California, San Francisco, California, USA; g Harbor Branch Oceanographic Institution, Florida Atlantic University, Center of Marine Ecosystems Health, Division of Marine Mammal Research and Conservation, Fort Pierce, Florida, USA; h Protect Wild Dolphins Alliance, Vero Beach, Florida, USA; i National Oceanic and Atmospheric Administration, National Ocean Service, Center for Coastal Environmental Health and Biomolecular Research, Charleston, South Carolina, USA; j Department of Environmental and Radiological Health Sciences, College of Veterinary Medicine and Biomedical Sciences, Colorado State University, Fort Collins, Colorado, USA; k Georgia Aquarium, Atlanta, Georgia, USA; l Division of Comparative Pathology, Miller School of Medicine, University of Miami, Miami, Florida, USA

## Abstract

The genome sequence of a papillomavirus was determined from fecal samples collected from bottlenose dolphins in the Indian River Lagoon, FL. The genome was 7,772 bp and displayed a typical papillomavirus genome organization. Phylogenetic analysis supported the bottlenose dolphin papillomavirus as being a novel type of Omikronpapillomavirus *1*.

## GENOME ANNOUNCEMENT

The family Papillomaviridae includes 48 genera of double-stranded DNA viruses (1, 2). Papillomaviruses (PVs) display a predilection for squamous epithelium and can cause benign or, less commonly, malignant neoplasia (3). In bottlenose dolphins (BDs), eight PVs have been characterized within the genera Omikronpapillomavirus (OmikronPV), Upsilonpapillomavirus (UpsilonPV), and Dyopipapillomavirus (4–9). Herein, we describe a novel type of OmikronPV1 sequenced from BD fecal samples.

Fecal swabs from 12 BDs were collected during a 2012 health assessment conducted under U.S. NMFS permit no. 14352 in the Indian River Lagoon (IRL), FL. Swabs were suspended in 1 ml phosphate-buffered saline and stored at −80°C. After thawing, the swabs were vortexed and centrifuged at 5,000 × *g* for 10 min. The clarified supernatant was filtered through a sterile 0.22-μm-pore-size filter and treated with a mixture of nucleases (10). Following the creation of two 6-sample pools, total nucleic acid was extracted using a commercial kit (10). For both pools, combined DNA and cDNA libraries were prepared as previously described (11, 12). Samples were sequenced using a V2 chemistry 500-cycle kit on an Illumina MiSeq sequencer.

*De novo* assembly of paired-end reads was performed in CLC Genomics Workbench. BLASTN searches of the resulting contigs, using the nonredundant nucleotide database maintained by the NCBI, recovered a 7,772-bp contig containing the full genome of a PV with highest sequence identity to previously reported cetacean OmikronPVs (4, 7, 8). Open reading frames (ORFs) were predicted using CLC Genomics Workbench, and the gene functions were determined using BLASTP searches. Six ORFs were predicted, with four early proteins (E1, E2, E4, and E6), two late proteins (L1 and L2), and a noncoding control region.

Seventy-six PV L1 nucleotide sequences were retrieved from the Papillomavirus Episteme resource (http://pave.niaid.nih.gov/) and aligned in TranslatorX (13) guided by amino acid translations with the MUSCLE alignment option. Maximum Likelihood phylogenetic analysis performed in IQ-TREE (14) supported the bottlenose dolphin papillomavirus (BDPV) within the OmikronPV1 clade. OmikronPV1 types have previously been determined from papillomatous lesions in BDs (7), harbor porpoise (Phocoena phocoena) (4), and Burmeister’s porpoise (Phocoena spinipinnis) (8). The L1 gene sequence of the BDPV was compared to those of all cetacean PVs and the type species of the other 46 PV genera using the Sequence Demarcation Tool (15). The BDPV displayed greatest nucleotide identity (75.8%) to OmikronPV1 (Tursiops truncatus papillomavirus 6 [TtPV6]) (7). Given that this is the ninth PV type described from a BD, it is referred to here as OmikronPV1 (Tursiops truncatus PV9 [TtPV9]).

An endpoint PCR assay targeting the OmikronPV1 (TtPV9) L1 gene sequence was designed to screen the 12 BD fecal samples. Three adult males, one displaying a genital papilloma noted during sampling, produced the expected 491-bp amplicons. Sequencing of the purified PCR products revealed that they were identical to OmikronPV1 (TtPV9). The reported OmikronPV1 (TtPV9) expands the genomic diversity of BDPVs. Further investigation into its prevalence and potential health impacts on free-ranging BDs is warranted.

### Accession number(s).

The sequence for OmikronPV1 (TtPV9) is available in GenBank under accession no. MG905161.
